# Measuring hope: psychometric properties of the children’s Hope Scale among South Sudanese refugee children

**DOI:** 10.1007/s10578-022-01327-6

**Published:** 2022-04-07

**Authors:** Janna Metzler, Yuan Zhang, Terry Saw, Cheng-Shiun Leu, Cassie Landers

**Affiliations:** Columbia University Mailman School of Public Health, 60 Haven Ave., B-2, 10032 New York, NY United States

**Keywords:** Hope, Resilience, Children, Humanitarian, Psychometrics

## Abstract

We investigated the psychometric properties of the Children’s Hope Scale among a sample of 1,118 South Sudanese refugee children (570 girls, 548 boys) aged 9 to 14 years displaced in Uganda. We assessed the underlying factor structure and model fit through exploratory and confirmatory factor analyses and measurement invariance by sex and developmental stage. Confirmatory factor analysis revealed good fit for a one-factor model with the error terms of items 1 and 3 and items 4 and 6 allowed to covary. There was no evidence of differential item functioning by group. Evidence from this study supports the use of a unidimensional model of hopefulness across groups and signifies the importance of confirming these properties for measures used to evaluate humanitarian interventions.

Trauma and the effects of conflict-related displacement profoundly impact the health and wellbeing of children and their families. The disruptions to social and communal mechanisms foundational to healthy development throughout childhood can result in lasting impairments. Intervening early to reduce the impacts of emergencies is critical to the way in which children engage, grow, develop, and thrive into productive and active members of any society.

Yet not all children experience lasting harm as a result of emergencies or experiences with conflict and forced displacement. Some children do well, are resilient (or exhibit a positive adaptive response to serious hardship), despite early and prolonged stress and adversity [1]. Resilient children have a range of protective experiences and adaptations which counterbalance the significant load of negative factors and adversity [2].

Research has increased our understanding of why some children do well and what factors are associated with positive outcomes for children. Much of this research has focused on establishing a core set of contributing factors including self-efficacy and adaptive capacities of the child, supportive adult-child relationships, and social and cultural support systems that often serve as a foundation for hope and stability [3]. Understanding the mechanisms that underpin resilience is critical in the designing of interventions to support children’s development and their diverse experiences and interactions with adversity.

However, the operationalization of resilience and measurement of its supportive factors remains challenging in humanitarian settings. Recent approaches have focused on examining the adaptive capacities of the child that may mitigate the effects of adverse events and facilitate the identification, navigation and attainment of resources [4]. Prior to this, research focused on the assessment of multi-level risk, promotive factors that work in opposition as a means of improving health and wellbeing, or protective factors that contribute to either a lessening of symptoms through moderation or reduction in the overall effects of risk [2, 6].

In creating or enhancing resilience, hope deserves attention. Hope mitigates the negative effects of trauma and adversity through one’s ability to develop pathways (*waypower*) towards reaching goals and apply agency (*willpower*) to these discovered pathways [7]. Hope is a dynamic process that drives well-being through this goal-directed focus and the ability to devise strategies to attain that goal [7]. There is a long-established science around hope documenting its predictive nature related to well-being and other important outcomes for children as well as validation efforts for its measurement for both adults and children [7].

The use of validated measures of hope provides opportunities for practitioners to design and implement effective programs for children that bolster different learning strategies that can be used to build hope and sustain its effects. One such measure, the Children’s Hope Scale (CHS), is a widely used tool measuring a child’s self-perception and level of belief about personal goal attainment [8]. Since its initial development, the CHS has been adapted for use in multiple countries, but few expressly for its use in humanitarian contexts [9–14]. There is still ongoing debate as to the factor structure with some studies noting a unidimensional structure of hopefulness and others, a two-factor structure signifying the *pathways* and *agency* that enables a child to evaluate feasible routes and the self-efficacy required to successfully navigate towards their goals [8]. Of note, one study amongst adolescents in the Western Cape Region of South Africa supported a unidimensional factor model of hope with the error terms of items 1 and 3 as well as items 4 and 6 allowed to covary (χ2 (7) = 35.692, p<.001; CFI=0.984; RMSEA=0.063; SRMR=0.023) [12]. This supposition was later tested in a population-based sample of children in South Africa to reveal a unidimensional factor model of hope with item 1 and 3 covariance [15]. The 2-factor structure hypothesized by Snyder and colleagues has been demonstrated in diverse populations [8, 9, 16]. Another study has found evidence to suggest a 2-factor structure with item 5 cross-loaded was a more appropriate fit for the data [17]. Overall, there is significant divergence in results across countries in which the measure is used. Furthermore, there is a dearth of psychometric evidence in humanitarian contexts to assess the effects of trauma and its related displacement and establish their contextual and cultural appropriateness amongst refugee and displaced populations. This study aims to further contribute to this by testing and validating the Children’s Hope Scale amongst a sample of South Sudanese refugee adolescents displaced in the West Nile Region of Uganda.

## Method

### Study Setting and Participants

 The current study uses baseline data from a randomized controlled trial (RCT) of the effectiveness of an enhanced package of services provided within a Child Friendly Space (CFS), a psychosocial intervention used to support the health and protection of children affected by conflict and displacement. A total of 1,118 children (570 girls, 548 boys) aged 9 - 14 years residing in the catchment areas surrounding the intervention sites participated in the study. Caregiver consent and child assent were taken prior to completion of an enumerator-administered questionnaire in Kakwa and South Sudanese Arabic. Interviews were conducted in semi-private locations near the primary residence of the child between May and July 2019. Intervention sites were in four villages within the Omugo Refugee Settlement within the West Nile Refugee Response. In addition to responses to the Children’s Hope Scale, participants were asked about their mental health, functional literacy and numeracy, and wellbeing. Survey administration took between 45 and 60 min.

### Instruments

The 6-item CHS developed by Snyder et al. [8], measures a child’s hopeful thinking and goal-directed beliefs, with higher sum scores indicative of more hope and goal-directed behavior. Scale items include: *I think I am doing pretty well* (chs1), *I can think of many ways to get the things in life that are most important to me* (chs2), *I am doing just as well as other kids my age* (chs3), *When I have a problem, I can come up with lots of ways to solve it* (chs4), *I think the things I have done in the past will help me in the future* (chs5), *Even when others want to quit, I know that I can find ways to solve the problem* (chs6) [8]. Items 2, 4 and 6 are intended to evaluate *pathways* thinking while items 1, 3, and 5 are meant to evaluate *agency* thinking. Response options are on a six-point scale ranging from *None of the time* (1) to *All of the time* (6).

Recent evidence of the validity and reliability of the three-factor, 12-item Child and Youth Resilience Measure (CYRM) suggests it may be effective to measure the acquisition of Individual, Relational and Contextual resources required to support and promote adolescent wellbeing [19 14]. Following confirmatory factor analysis of the CYRM, this 12-item measure of resources available to individuals to strengthen their resilience demonstrated the best fit for these data (χ2 (51) = 183.04, p<.001; RMSEA=0.068 (90% CI = 0.058 – 0.079); CFI=0.928; TLI = 0.907; SRMR=0.052). Response options are on a 5-point Likert scale with responses ranging from *Not at all* (0) to *A lot* (5).

### Data Analysis

Descriptive statistics, data management, and correlation analysis were performed using STATA version 14.2. Using data from the first timepoint, exploratory factor analysis (EFA) was conducted in Mplus version 8.1 to explore the dimensionality of the construct of hope, determining the best model fit for the emergent factor structure. Overall, scale items had less than 2% missing data. The full sample was randomly divided into two equal datasets for use in first the exploratory analysis (EFA, validation dataset) and the second for confirmatory factor analysis (CFA, confirmation dataset) of the CHS items. Threshold and parameter estimation were based on weighted least-squares with mean and variance adjustment (WLSMV), given the ordered categorical items of the measure. We assessed the appropriateness of the data for EFA by the total sample size being greater than 300 [18], the Kaiser-Meyer-Olkin test for Sampling Adequacy (KMO) > 0.6 [19], and a significant result (p < .05) for Bartlett’s Test of Sphericity indicating variable interrelationships [20]. We used oblique rotation (promax) to assess correlation matrices, retaining factor correlations of ± 0.32 or greater [19, 21]. This indicates 10% or more overlap in variance among factors.

Confirmatory factor analyses were then conducted using the second confirmation dataset. The selected EFA model was compared to other measurement models represented in the literature: (1) a unidimensional factor structure (Model 1) in which all indicators load freely onto a single latent variable of hope, (2) a unidimensional factor structure (Model 4) was specified in which the error terms of item 1 varies with item 3 and the error terms of item 4 varies with item 6 [12], (3) a two-factor structure (Model 2) in which items 1, 3 and 5 load onto a latent variable of *agency*, and in which items 2, 4 and 6 load onto a latent variable of *pathways* [8], (4) a two-factor structure (Model 3) similar to Model 2 with item 5 cross-loaded [17], and a unidimensional factor structure (Model 6) with the error terms of item 4 and 6 covarying [9]. All measurement error in the 2-factor models were presumed to be uncorrelated, and the first loading of each factor was fixed to 1.0.

CFA was examined using weighted least squares estimation using Mplus v. 8.1 [23]. The overall fit for each factor model was assessed with the following criteria: the root mean square error of approximation (RMSEA) < 0.06, standardized root-mean-square residual (SRMR) < 0.08, non-negative residual estimates, comparative fit index (CFI) > 0.95, Tucker-Lewis index (TLI > 0.95, and nonsignificant Chi-square goodness of fit tests. We examined the scree plots for points of inflection. The best fitting EFA model was selected based on the statistically appropriate model and overall interpretability of the model. Additionally, model fit was also evaluated using Akaike information criterion (AIC) [24] and Bayesian information criteria (BIC) [25], with lower values indicating a more parsimonious fit.

After determining the overall best fitting model, configural, metric, and scalar invariance was tested by age (pre-adolescence vs. early adolescence) and sex (girls vs. boys). Configural invariance was tested to examine if the same set of factors is present across these similar groups. Metric invariance was tested to determine if the factor loadings are equal across these groups; an indication of the magnitude of correlation between items assigned to the underlying trait. Scalar invariance was tested to determine if there were any systematic differences in individual response patterns due to group membership.

Multigroup modeling was examined using robust maximum likelihood estimation using Mplus v. 8.1 [23]. Nested model comparisons were conducted using the −2LL rescaled difference test. In the configural invariance model, the factor mean was fixed to 0 and the factor variance was fixed to 1 for identification within each group. Factor loadings, intercepts and residual variances were free across groups. A residual covariance between items 1 and 3 and items 4 and 6 were also estimated in each group as suggested by previous results. In the metric invariance model, the equality of the unstandardized item factor loadings across groups was examined. The factor variance was fixed to 1 in girls but was freely estimated in boys, and the factor means in both groups were fixed to 0. All factor loadings were constrained to be equal across groups. All intercepts and residual variances (and the residual covariance between items 1 and 3 and items 4 and 6) were allowed to vary across groups. The scalar model examined the equality of the unstandardized item intercepts across groups. The factor mean and variance were fixed to 0 and 1, respectively, but residual variances were free across groups. All residual variances (and the residual covariance between items 1 and 3 and items 4 and 6) were still allowed to differ across groups.

## Results

There were 1,118 participants (51.0% girls) between the ages of nine and 14 years (mean = 11.61 years, SD = 1.64) in the full sample. Of this, 559 were randomly allocated to each half sample. Descriptive statistics of the full sample and each half sample are displayed in Table 1.

**Table 1:** Descriptive Statistics

	Complete Sample	EFA	CFA
	(N=1116)	(N= 558)	(N= 558)
	N (%) / Mean (SD)		
**Age (years)**	11.62 (1.6)	11.62 (1.7)	11.61 (1.6)
**Female**	569 (51.0)	283 (50.7)	286 (51.3)
**Catchment Area**			
**1**	61 (5.5)	34 (6.1)	27 (4.8)
**2**	384 (34.4)	183 (32.8)	201 (36.0)
**3**	91 (8.2)	40 (7.2)	51 (9.1)
**4**	576 (51.6)	298 (53.4)	278 (49.8)
**Other**	4 (0.3)	3 (0.5)	1 (0.2)
**Ethnicity**			
**Bari**	922 (82.6)	461 (82.6)	461 (82.6)
**Other minority groups**	194 (17.4)	97 (17.4)	97 (17.4)
**Religion**			
**Christian**	1088 (97.5)	546 (97.9)	542 (97.1)
**Muslim**	20 (1.8)	10 (1.8)	10 (1.8)
**Other**	8 (0.7)	2 (0.3)	6 (1.1)
**Displaced by conflict**	1056 (94.6)	529 (96.9)	527 (96.0)
**Attends school**	1,100 (98.6)	550 (98.6)	550 (98.6)

EFA exploratory factor analysis, CFA confirmatory factor analysis

### Exploratory Factor Analysis

The EFA produced eigenvalues of 3.08, 1.03, 0.63, 0.52, 0.42, and 0.32. Two of these eigenvalues were greater 1.0 [26] and explained 41.1% of the total variance in the data. Examination of the scree plot revealed one clear inflection point at two factors.

Table 2 displays the item and factor loadings from the exploratory factor analysis for the final model tested in CFA. The highest item loading was aligned and retained for each factor. Based on comparative fit statistics and interpretability, a two-factor model was retained for the subsequent CFA. In this model, items 1 through 3 loaded to factor one and items 4 through 6 loaded to factor two.

**Table 2:** Factor Loadings of the CHS Among the Random EFA Subsample

	One-Factor Model	Two-Factor Model	
**Item Content**		**Factor 1**	**Factor 2**
CHS1: I think I am doing pretty well.	0.64	0.76	0.32
CHS2: I can think of many ways to get the things in life that are important to me.	0.62	0.62	0.47
CHS3: I am doing just as well as other kids my age.	0.75	0.78	0.50
CHS4: When I have a problem, I can come up with lots of ways to solve it.	0.68	0.48	0.74
CHS5: I think the things I have done in the past will help me in the future.	0.60	0.56	0.50
CHS6: Even when others want to quit, I know that I can find ways to solve the problem.	0.65	0.42	0.84

### Confirmatory Factor Analysis

The EFA two-factor model was evaluated among five existing models identified in the literature. Each of the overall goodness-of-fit indices suggested poor global fit to the data (see Table 3). Overall, the one-factor model of hope with the error terms of items 1 and 3 and items 4 and 6 allowed to covary fit the data well: χ2 (7) = 36.83, p<.001; RMSEA=0.087 (90% CI = 0.061 – 0.116); CFI=0.987; TLI = 0.971; WRMR=0.647.

**Table 3:** Fit Indices for Models of Children’s Hope Evaluated in CFA (using WLSMV)

Model	# Free Parameters	χ2	df	p-value	CFI	TLI	RMSEA Estimate	RMSEA Lower CI	RMSEA Higher CI	RMSEA p-value	WRMR
1	36	254.06	9	<0.0001	0.893	0.821	0.221	0.198	0.245	<0.0001	1.82
2	37	182.30	8	<0.0001	0.924	0.857	0.198	0.173	0.223	<0.0001	1.53
3	38	138.94	7	<0.0001	0.942	0.876	0.184	0.158	0.211	<0.0001	1.28
4	38	37.70	7	<0.0001	0.987	0.971	0.089	0.062	0.117	<0.0001	0.65
5	37	94.28	8	<0.0001	0.962	0.929	0.139	0.115	0.165	<0.0001	1.08
6	37	94.28	8	<0.0001	0.962	0.929	0.139	0.115	0.165	<0.0001	1.08

Model 1 = 1-factor (hopefulness); Model 2 = 2-factor (pathways, agency) [8]; Model 3 = 2-factor with cross-loading of item 5 [17]; Model 4 = 1-factor with co-varying error terms on items 1 and 3 and items 4 and 6 [12]; Model 5 = 2-factor EFA; Model 6 = 1-factor with co-varying error terms on items 4 and 6 [15].

### Convergent validity and internal consistency

Results showed significant correlations in the expected directions for each of the factors of the Child and Youth Resilience Measure (Individual r = .41, p<.001; Relational r = .18, p<.001; Contextual r = .17, p<.001). For the total scale, the results showed adequate levels of internal consistency (α = 0.79).

*Measurement Invariance by Sex and Developmental Stage (middle childhood, 9-11 years vs. early adolescence, 12-14 years)*.

For sex, the configural model fit well (see Table 4). Parameter constraints were then applied to see whether there were decreases in model fit resulting from measurement or structural non-invariance. The metric invariance model fit well and revealed no significant decrease in fit relative to the configural model, −2ΔLL(5) = 7.48, p = .19. No points of localized strain among the constrained loadings were detected. This suggests that the same latent factor was being measured in each group. The scalar invariance model fit well (see Table 4) and did not result in a significant decrease in fit relative to the metric invariance model, −2ΔLL(5) = 2.96, *p* = .71. No points of localized strain among the constrained loadings were detected. This suggests that the observed differences in item means between groups were due to factor mean differences only.

**Table 4:** Measurement Invariance by Gender and Age group

Model	Reference model	χ2	χ2 Scale Factor	df	p-value	RMSEA	CFI	TLI	SRMR	AIC	BIC
Gender											
1. Configural		22.42	1.25	14	0.0704	0.046	0.987	0.973	0.027	11460.15	11633.13
2. Metric	1	25.84	1.20	19	0.1348	0.036	0.990	0.984	0.034	11453.25	11604.60
3. Scalar	2	31.38	1.16	24	0.1432	0.033	0.989	0.986	0.037	11448.62	11578.35
Age Group											
1. Configural		33.69	1.24	14	0.0023	0.071	0.971	0.939	0.032	11455.75	11628.72
2. Metric	1	42.13	1.20	19	0.0017	0.066	0.966	0.947	0.048	11454.33	11605.68
3. Scalar	2	48.53	1.16	24	0.0022	0.061	0.964	0.955	0.052	11450.01	11579.75

Note. df = degrees of freedom; RMSE = root mean square error of approximation; CFI = comparative fit index; TLI = Tucker-Lewis index; SRMR = standardized root mean square residual; AIC = Akaike Information Criterion; BIC = Bayesian information criterion.

For developmental stage, the configural, metric, and scalar models fit well (see Table 4). The metric invariance model revealed no significant decrease in fit relative to the configural model, −2ΔLL(5) = 4.36, p = .50. No points of localized strain among the constrained loadings were detected. This suggests that the same latent factor was being measured in each group. The scalar invariance model fit well did not result in a significant decrease in fit relative to the metric invariance model, −2ΔLL(5) = 6.78, *p* = .24. No points of localized strain among the constrained loadings were detected. This suggests that the observed differences in item means between groups were due to factor mean differences only.

## Discussion

Using data from an RCT of Child Friendly Space interventions, this study aimed to explore hope amongst a sample of South Sudanese refugee children residing in refugee resettlement areas in the West Nile region of Uganda. We assessed the factor structure, measurement invariance, and validity of the Children’s Hope Scale to document its contextual and cultural appropriateness amongst this displaced population and explore its ability to distinguish levels of hope amongst different subgroups.

Upon initial exploratory testing, the results suggested a two-factor model fit these data best. However, when evaluated against other existing models from the literature, a unidimensional model with error covariances between items 1 and 3 and items 4 and 6 was found to fit best. This finding resonates with previous research conducted by Savahl et al. amongst a sample of adolescents in the Western Cape Region of South Africa [12]. The use of a one-factor model suggests that children in this sample conceptualize hope as a whole and do not discriminate between agency and pathways thinking. Recent research exploring hope amongst Chinese and American adolescents illuminates the cross-cultural diversity of conceptualization of the construct along the collectivistic-individualistic continuum, with those having a more collectivist orientation aligned with a single construct of hopefulness [27]. The recommendation of the utility of a total CHS score has also been proposed in similar studies to ours when the factors are highly correlated and theorized to be indistinguishable from each other [12, 17].

We used multigroup modeling to test measurement invariance, suggesting that observed differences in item means were due to factor mean differences only and not related to sex of the child or whether the child was in middle childhood or the early adolescent period of development. This finding suggests that girls and boys as well as younger and older children conceptualize hope in a similar way, further providing evidence of the utility of this measure for determining differential impacts of interventions by sex and developmental stage.

This study has limitations that should be considered. First, a large sample was collected from catchment areas surrounding the intervention locations but was not representative of the entire refugee population in the West Nile region. As a result, findings may be limited with respect to generalizability. Second, this study examined invariance only across sex and developmental stage. Other variables, such as ethnicity, displacement, and time, should be explored further for their influence on the measurement of hope.

Hope and resilience are fundamental to the continuing development and health of children in humanitarian settings. This study provides some of the first evidence to support the use of the CHS in measuring the hopefulness of refugee children and further understanding the effectiveness of interventions designed to bolster hope and resilience. However, more needs to be done to have a comprehensive understanding of the mechanisms of hope and its functionality across time. Further efforts should be made to explore measurement invariance of hope across diverse conflict and crisis-affected populations. Finally, given the role of hope in buffering stress and promoting resilient trajectories in children, practitioners should focus their efforts on developing and strengthening the capabilities that underlie hope by age-appropriate activities that build skills to cope with, adapt to, and mitigate adversity. Suggested activities could include strategies to (a) identify and prioritize solution-specific goals and provide strategies for creating an image picture of what is most important; (b) breakdown the goals—especially long-term ones—into a series of small steps that can be acknowledged and celebrated; (c) develop problem-solving skills to identify alternative paths and creative ways to overcome obstacles; (d) share stories that illustrate how others have overcome adversity to reach goals and draw on personal and family stores of success; and e) encourage positive “can do” thoughts when confronting obstacles. The science of hope can and must be incorporated into humanitarian programs for children.

## Summary

Results from confirmatory factor analysis revealed a good fit for a one-factor model with the error terms of items 1 and 3 and items 4 and 6 allowed to covary. There was no evidence of differential item functioning by sex or developmental stage. Implications of these findings include the continued use of the Children’s Hope Scale and valid appraisal of the level of hopefulness amongst crisis-affected girls and boys from middle childhood to early adolescence. Given the diverse origins of the mechanisms of hope and its pathways towards resilient outcomes for children, it would be important to explore measurement invariance of hope across diverse conflict and crisis-affected populations, its functionality across time, and examine and test interventions that aim to develop and strengthen the capabilities that underlie hope.
